# Systolic Anterior Motion of the Mitral Valve Post-Heart Transplant Secondary to an Unusual Mechanism

**DOI:** 10.7759/cureus.74745

**Published:** 2024-11-29

**Authors:** Tara Lewandowski, Nirav Mathur, Nikita Ovtchinnikov, Ben Brakke, Venkat Mangunta, Christian M Renwick

**Affiliations:** 1 Department of Anesthesia, Southeastern University, Lakeland, USA; 2 Department of Anesthesiology, Saint Lukes Hospital of Kansas City, Kansas City, USA; 3 Department of Anesthesiology, Beth Israel Deaconess Medical Center, Boston, USA; 4 Department of Anesthesia and Critical Care, Mayo Clinic, Rochester, USA; 5 Division of Cardiothoracic Anesthesia, Department of Anesthesiology, Lehigh Valley Health Network, Charlottesville, USA; 6 Department of General Surgery, University of Virginia, Charlottesville, USA

**Keywords:** cardiac surgery, mitraclip, orthotopic heart transplant, systolic anterior motion of the mitral valve, transesophageal echocardiogram

## Abstract

In this case report, we discuss the critical interdependence of structure and function in demonstrating systolic anterior motion (SAM) of the mitral valve after repeat heart transplantation, where residual apical tissue of the explanted heart remained in place. The resulting conformational changes led to anterior displacement of the mitral valve and persistent SAM.

## Introduction

Systolic anterior motion (SAM) of the mitral valve (MV) is an uncommon cause of left ventricular outflow obstruction, where the dynamic systolic movement of the anterior leaflet of the MV transiently obstructs outflow of the left ventricle (LV) [1]. Still, it is an important diagnosis to include within the differential for patients who demonstrate shock physiology after heart transplant. This case report illustrates the critical interdependence of structure and function in demonstrating SAM of the MV after repeat heart transplantation, where residual apical tissue of the explanted heart remained in place. The resulting conformational changes led to anterior displacement of the MV and persistent SAM. Furthermore, this case report demonstrates a unique application of the MitraClip device (Abbott Vascular, Chicago, IL, United States) for patients demonstrating SAM physiology and are otherwise unsuitable surgical candidates.

## Case presentation

A 66-year-old male presented for repeat orthotopic heart transplantation (OHT) secondary to severe heart failure with preserved ejection fraction. His prior OHT in 2013 was complicated by an undersized heart, constrictive pericarditis requiring pericardiectomy, and progressive fibrosis resulting in a restrictive filling pattern. Over the intervening years, his symptoms progressed to shortness of breath at rest, abdominal distension, and New York Heart Association (NYHA) Functional Capacity IV, Objective Assessment D.

Preoperative right heart catheterization for his repeat OHT demonstrated pulmonary arterial (PA) pressures of 51/24/35 mmHg, pulmonary capillary wedge pressure of 26 mmHg, and a cardiac index (CI) of 1.88 L/min/m^2^. Preoperative echocardiography demonstrated grade III diastolic dysfunction. An intra-aortic balloon pump was placed in the setting of elevated filling pressures and decreased cardiac output. Given his progressive low cardiac output state, he was urgently re-listed for repeat OHT as a status 1A.

**Figure 1:**
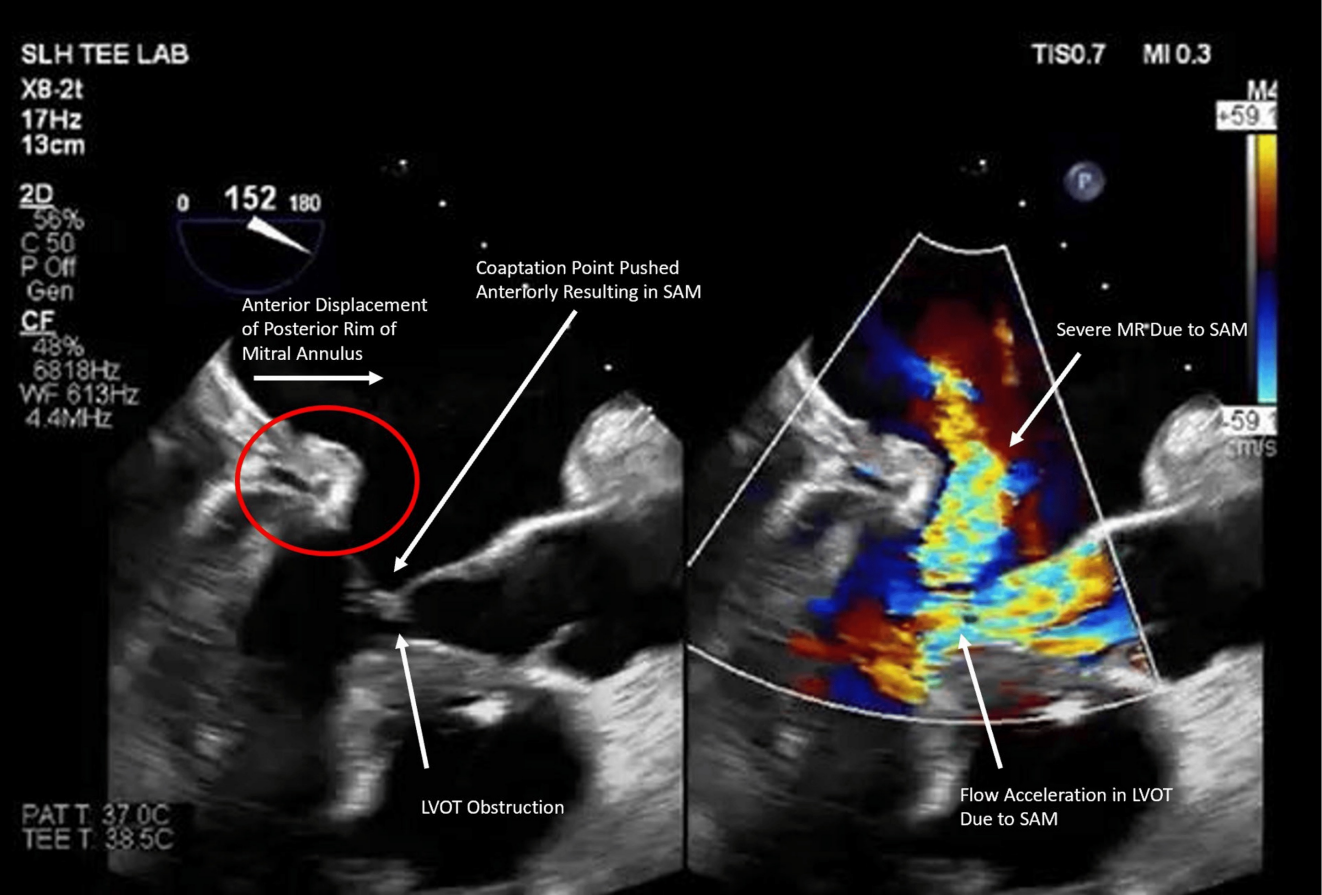
Transesophageal echocardiogram. Transesophageal echocardiogram with color Doppler (right) showing severe MR secondary to valvular SAM post-heart transplant. The posterior mitral annulus is anteriorly displaced secondary to reflected tissue (red circle), causing the coaptation point to be similarly displaced anteriorly and the development of LVOT obstruction. SAM, systolic anterior motion; LVOT, left ventricular outflow tract; MR, mitral regurgitation

Intraoperative transesophageal echocardiography (TEE) after placement of the new heart allograft demonstrated adequate right ventricular function, a hyperdynamic LV with an estimated EF of 70%, and SAM of the MV with a posteriorly directed mitral regurgitation (MR) jet. Serial echocardiography in the intensive care unit between postoperative days 1 and 5 demonstrated persistent SAM with a low cardiac output state. Medical management of this patient's SAM consisted of intravascular volume expansion and increasing afterload while minimizing inotropic agents. PA pressures were documented up to 90/40 mmHg. The post-transplant persistent SAM was suspected to have resulted from residual tissue of the explanted heart (from this patient's first OHT), distorting the normal anatomy of the new heart. This led to the anterior displacement of the posterior rim of the MV, causing the severe MR jet, as shown in Figure 1.

**Figure 2:**
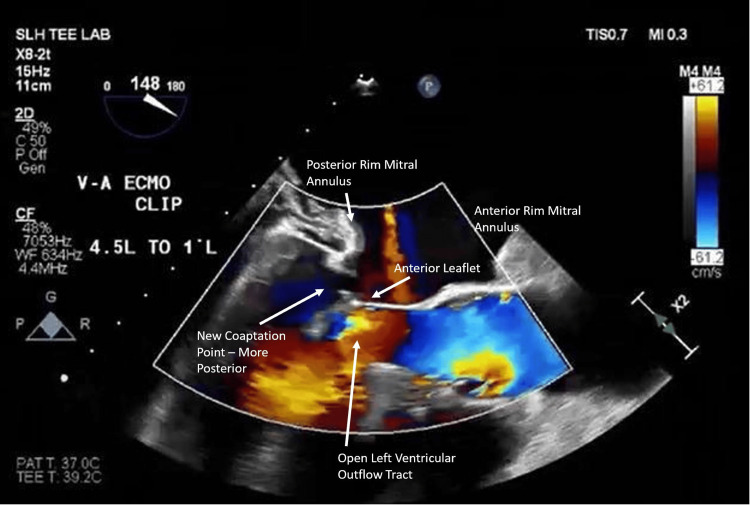
Post-MitraClip transesophageal echocardiogram. Transesophageal echocardiogram with color Doppler demonstrating resolution of severe MR post-MitraClip deployment. The MitraClip procedure did not affect the positioning of the posterior mitral annulus, but instead brought the anterior leaflet and coaptation point more posteriorly, resolving the left ventricular outflow tract obstruction. MR, mitral regurgitation

After an in-depth discussion between cardiothoracic surgery, cardiology (heart failure, structural, and echocardiography teams), critical care, and cardiovascular anesthesiology, we determined that he was at prohibitive risk for a fourth sternotomy given his multiorgan failure, coagulopathy, and the difficult dissection during transplant due to high scar tissue burden. One week post-operatively, he was taken for transcatheter mitral repair with a MitraClip NTW device at the A2/P2 scallops to “pull” the leaflet coaptation point back to the midline. Unfortunately, the patient required extracorporeal membrane oxygenation (ECMO) cannulation due to respiratory failure, with severe hypoxia, when laid supine for the MitraClip procedure. After a successful transseptal puncture, the MitraClip captured approximately 6 mm of the P2 scallop and the leading edge of the A2 scallop, eliminating SAM and the MR jet (Figure 2). After MitraClip deployment, our patient’s liver enzymes and lactate improved, allowing us to wean pressors and decannulate from ECMO four days later. Immediately after decannulation, his CI was 3.2 L/min/m^2^ and PA pressures were 40/22 mmHg.

Unfortunately, one day after ECMO decannulation, the patient developed worsening lactic acidosis likely related to mesenteric ischemia and passed away due to severe metabolic acidosis and multiorgan failure.

No patient-identifying information was provided, and all imaging is depicted with no patient identifiers. Therefore, this case report was exempt from informed consent.

## Discussion

**Figure 3:**
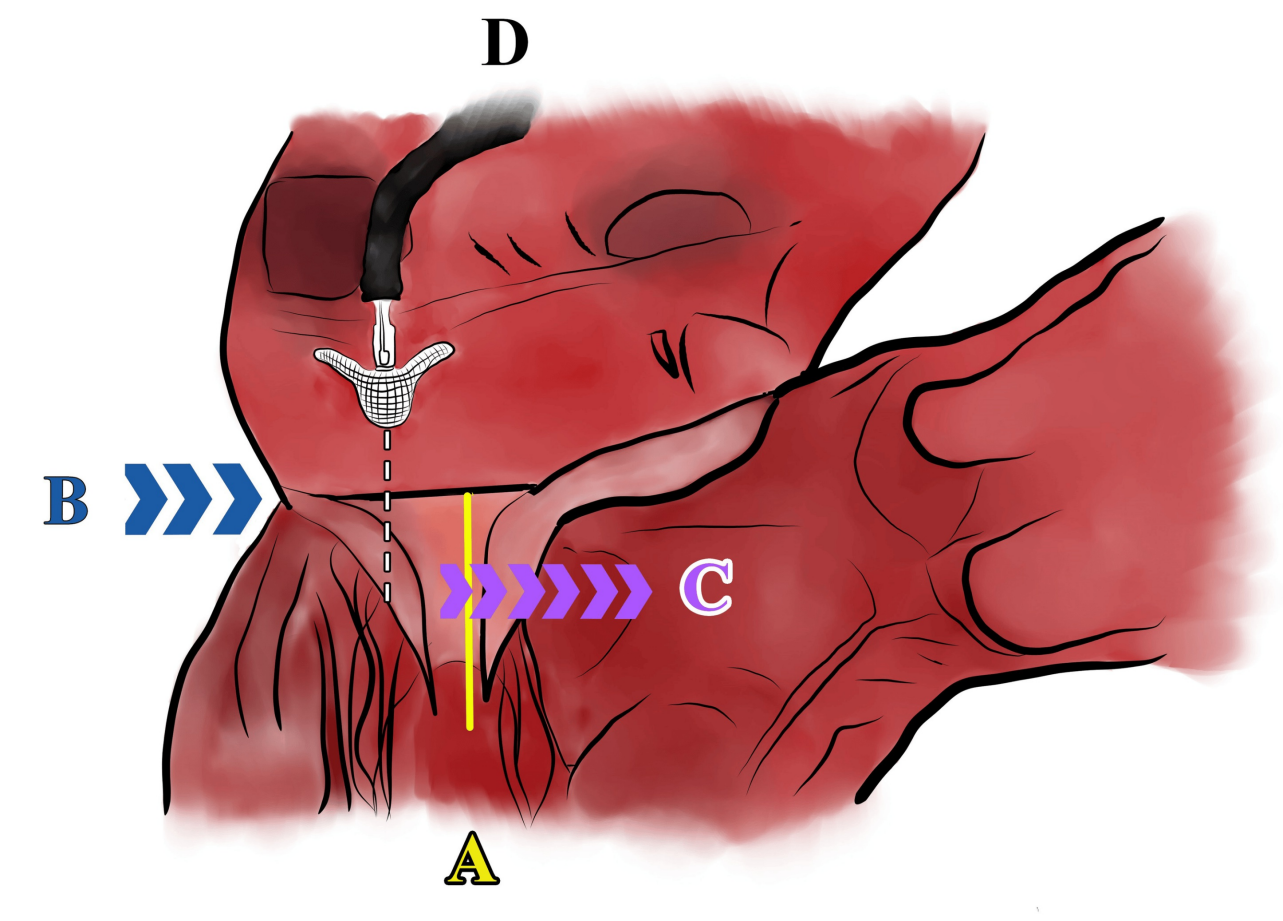
Structural changes leading to SAM and resolution with MitraClip deployment. (A) Normal MV plane of coaptation. (B) Posterior rim of the MV annulus is pushed anteriorly. (C) The MV apparatus is pushed toward the LVOT moving the coaptation point anteriorly resulting in SAM. (D) With deployment of the MitraClip, the A2 and P2 scallops of the MV are captured, restoring the normal coaptation point of the MV. This figure is an original drawing by the authors. SAM, systolic anterior motion; LVOT, left ventricular outflow tract; MV, mitral valve

SAM describes the dynamic systolic movement of the anterior leaflet of the MV toward the left ventricular outflow tract (LVOT), transiently obstructing the LV outflow. Classically, the primary hypothesized mechanism of SAM has been the Venturi effect-reduced pressure caused when fluid flows through a narrow area at high velocity. The low-pressure area created by high-velocity flow then lifts the anterior leaflet of the MV toward the septum and LVOT [1]. Sherrid et al. described early onset SAM in hypertrophic cardiomyopathy (HCM) patients where there is normal flow velocity flow through the LVOT at the initiation of SAM [2]. This contradicts the premise that Venturi forces are primarily responsible as they require high-velocity flow, resulting in movement of the anterior leaflet perpendicular to the direction of flow [2]. Therefore, while Venturi forces act as velocity increases, drag forces predominate at lower velocities, pushing the anterior leaflet in the direction of flow (Figure 3) [2]. In non-HCM patients, SAM can be characterized as chordal or valvular, with valvular SAM associated with higher LVOT pressure gradients and more significant MR. It is important to remember that not all dynamic LVOT obstruction is due to HCM and to retain a high index of suspicion for SAM due to other etiologies [1,3]. In our patient, the LVOT obstruction appeared consistent with valvular SAM given the higher LVOT pressure gradient (64 mmHg) and severe MR.

This rare mechanism of SAM development posed a problematic clinical scenario for the multidisciplinary cardiovascular team. Due to the significant adhesions and scar tissue from two prior sternotomies, a portion of the apex of the recipient’s heart had to be left in place. In such bi-atrial techniques, the right and left atrial cuffs are left in place to facilitate the implantation of the orthotopic heart. This tight fit, combined with a bi-atrial transplant technique leading to the left atrial suture line reflecting anteriorly and displacing the mitral annulus towards the LVOT, led to marked anterior displacement of the MV coaptation point (Figure 3). These structural changes, low systemic vascular resistance, increased chronotropy post-transplant, and fluctuating volume status set the stage for early SAM.

## Conclusions

This case illustrates the critical interdependence of structure and function with alterations in the geometric structure of the heart resulting in SAM after repeat OHT. In patients undergoing OHT, it is imperative to monitor the cardiac structure with TEE during transplantation to identify changes that may alter allograft function. Timely involvement of a multidisciplinary team capable of high-fidelity communication, structural and surgical interventions, and management of patients in multi-organ failure allow for early recognition of unusual mechanisms, leading to clinical decline. In such situations, where patients may not be candidates for surgical interventions and fail medical management, a lower threshold for endovascular interventions is warranted.
